# Project HELP: a study protocol to pilot test a shared decision-making tool about treatment options for patients with hepatitis C and chronic kidney disease

**DOI:** 10.1186/s40814-018-0251-2

**Published:** 2018-02-21

**Authors:** M. C. Politi, N. George, T. Li, K. M. Korenblat, K. J. Fowler, C. Ho, A. Liapakis, D. Roth, J. Yee

**Affiliations:** 1 Division of Public Health Sciences, Department of Surgery, Washington University in St. Louis School of Medicine, 660 S. Euclid Ave., Campus Box 8100, St. Louis, MO 63110 USA; 2 Department of Internal Medicine, Division of Nephrology, Washington University School of Medicine, 660 S. Euclid Ave., Campus Box 8129, St. Louis, MO 63110 USA; 3 Department of Internal Medicine, Division of Gastroenterology, Washington University School of Medicine, 660 S. Euclid Ave., Campus Box 8124, St. Louis, MO 63110 USA; 4 The Voice of the Patient Inc., 908 South Cambridge Ave., Elmhurst, IL 60126 USA; 5 California Pacific Medical Center, 2340 Clay Street, 3rd floor, San Francisco, CA 94115 USA; 6 Department of Internal Medicine Section of Digestive Disease, Yale University School of Medicine, 333 Cedar St., PO Box 208019, New Haven, CT 06520 USA; 7 Katz Family Division of Nephrology and Hypertension, University of Miami Miller School of Medicine, 120 NW 14th St. Room 813, Miami, FL 33136 USA; 8 Division Head of Nephrology, Hypertension & Transplant, Henry Ford Hospital and Medical Center, 2799 West Grand Blvd, CFP-514, Detroit, MI 48202-2689 USA

**Keywords:** Hepatitis C, Chronic kidney disease, Patient centered, Decision aids, Decision support, Health communication

## Abstract

**Background:**

Recent advances in treatment have given patients with chronic kidney disease (CKD) access to safer and more effective medications to treat comorbid hepatitis C virus (HCV) infection. Given the variety and complexity of treatment options that depend on patients’ clinical characteristics and personal preferences, education and decision support are needed to prepare patients better to discuss treatment options with their clinicians.

**Methods:**

Drawing on International Patient Decision Aids Standards guidelines, literature reviews, and guidance from a diverse expert advisory group of nephrologists, hepatologists, and patients, we will develop and test a HCV and CKD decision support tool. Named *Project HELP* (*Helping Empower Liver and kidney Patients*), this tool will support patients with HCV and CKD during decisions about whether, when, and how to treat each illness. The tool will (1) explain information using plain language and graphics; (2) provide a step-by-step process for thinking about treating HCV and CKD; (3) tailor relevant information to each user by asking about the individual’s stage of CKD, stage of fibrosis, prior treatment, and comorbidities; (4) assess user knowledge and values for treatment choices; and (5) help individuals use and consider information appropriate to their values and needs to discuss with a clinician. A pilot study including 70 individuals will evaluate the tool’s efficacy, usability, and likelihood of using it in clinical practice. Eligibility criteria will include individuals who understand and read English, who are at least 18 years old, have a diagnosis of HCV (any genotype) and CKD (any stage), and are considering treatment options.

**Discussion:**

This study can identify particular characteristics of individuals or groups that might experience challenges initiating treatment for HCV in the CKD population. This tool could provide a resource to facilitate patient-clinician discussions regarding HCV and CKD treatment options.

## Background

Hepatitis C virus (HCV) infection is a chronic, debilitating disease that affects 170 million people globally and about 3.9 million individuals in the USA [1]. This public health issue is the most common blood-borne illness in the USA [2] and is especially prevalent among those with chronic kidney disease (CKD). The estimated prevalence of HCV in hemodialysis patients in the USA is 8.6% [2], almost five times greater than in the general US population [3]. Individuals with untreated HCV can develop serious complications including cirrhosis, liver failure, and/or hepatocellular carcinoma [4].

Among patients with CKD, the presence of HCV can significantly affect their quality of life and health outcomes. Patients with CKD who also have chronic HCV infection may have an increased risk of death, hospitalization, loss of kidney function, and kidney transplant failure [4–10]. HCV infection has been associated with lower quality of life in patients on dialysis and may be particularly adverse to patients’ mental health [11]. The goal of therapy for chronic HCV infection is a sustained virologic response (SVR) where the disease is undetectable 12 to 24 weeks following discontinuation of therapy [12]. SVR reduces the chance of cirrhosis of the liver, liver cancer, the need for liver transplantation, and overall death rates among patients with CKD [13–16]. As a result, national and international guidelines suggest that HCV-infected patients with CKD should consider antiviral treatment for their HCV [17].

In the past, few patients with CKD received treatment for their HCV infection despite the potential benefits [18]. Early medications for HCV infection included interferon-based therapies in combination with ribavirin. These drugs were not always effective, eliminating HCV from only 14 to 63% of patients [19]. Many patients experienced severe side effects and reduced their doses or discontinued treatment on these medications [20, 21]. Because side effects such as anemia were amplified in patients with CKD, treating HCV in CKD patients was especially challenging, and many patients with CKD and their clinicians felt that the potential benefits of treatment might not outweigh the potential risks. As a result, as few as 1% of HCV+ CKD patients were prescribed antiviral medications [2].

Over the past 4 years, oral direct-acting antiviral (DAA) agents, molecules that target specific nonstructural proteins of the virus and interrupt HCV viral replication have become available as treatment options for patients with HCV infection [22]. DAA regimens have a higher SVR (> 95%) and relatively few toxicities compared to previous treatments [23, 24]. They also have easier dosing regimens and fewer interactions with other medications used to treat patients with comorbidities [25]. There are now DAA regimens FDA approved for patients with a creatinine clearance < 30 ml/min. Studies suggest that patients with severe renal impairment tolerate treatment with DAAs well, and have many positive outcomes from these medications [26, 27].

With these advances in therapeutic options, patients with both HCV and CKD now face different complex decisions. The primary decision is the timing of HCV therapy, especially for renal-impaired patients [14, 17]. Successful anti-viral therapy can halt the progression of liver fibrosis, reduce the risk of hepatocellular carcinoma, improve quality of life, and even potentially reduce the risk of progression of CKD [22, 28]. However, delaying therapy may allow patients to consider acceptance of a kidney allograft from a HCV viremic donor and allow for early access to transplant, reduced time on dialysis, and a reduction in the associated mortality of renal replacement therapy. Specifics of the DAA regimens (i.e., dosing, labeling in end-organ disease or organ damage due to disease progression, and cost) as well as drug-drug interactions need to be considered [17, 22].

Occasionally, among those with multiple comorbidities and severe CKD, patients must consider whether HCV treatment would improve their quality of life. Patients and clinicians should weigh these complexities in treatment options and timing; tailored decision tools may help guide patients and clinicians through this decision process. Clinicians often use management paradigms to guide their assessment for each patient’s case [29]. With the recent increase in the use of kidneys from HCV-infected donors for transplants, there is a potential to generate positive health outcomes for vulnerable populations with advanced CKD and HCV [30]. However, not all patients are good candidates for kidney transplantation.

Patients’ subjective preferences for the tradeoffs between potential benefits and harms of treatment options can impact choices. Patients’ insurance coverage and potential out-of-pocket costs can also affect their access and adherence to treatment [31]. To make treatment decisions, patients must balance each of these factors, as well as advice from multiple clinicians, including nephrologists, hepatologists, and sometimes a primary care provider. Given the variety and complexity of treatment options that depend on patients’ clinical characteristics and personal preferences, interventions are needed to better prepare patients to discuss options with clinicians.

Patient decision aids are designed to help patients understand complex health options and take an active role in decision-making. Decision aids differ from educational materials because of their detailed, precise, and personalized focus on options, outcomes, probabilities, and patients’ values. Decision aids have been shown to reduce decisional conflict, increase patient’s knowledge of treatment options, lower decision regret, increase patient involvement in decisions, reduce patient indecision concerning treatment, and increase the probability that treatment decisions will be consistent with patients’ values [32].

**Fig. 1:**
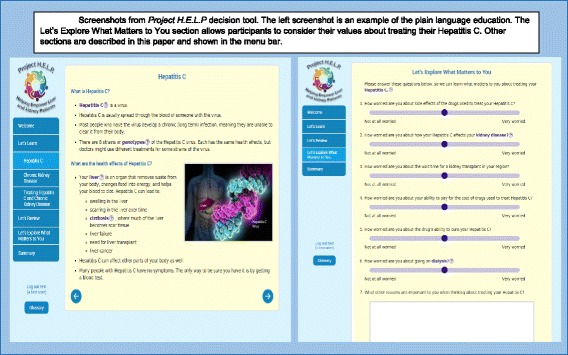
Screenshots from Project HELP decision tool. The left screenshot is an example of the plain language education. The Let’s Explore What Matters to You section allows participants to consider their values about treating their hepatitis C. Other sections are described in this paper and shown in the menu bar

This study, called *Project HELP* (Helping Empower Liver and kidney Patients), will develop and test a web-based decision aid to support patients with HCV and CKD during decisions about whether, when, and how to treat each illness. Patients will also have the opportunity to learn about their hepatitis C and kidney disease, initiate thought about what matters most to them and choose a treatment plan for their liver and kidney disease that works best for them. This decision aid will:

1. Explain information using plain language and graphics [see Fig. 1] and provide a step-by-step process for thinking about treating HCV and CKD
2. Tailor relevant information to each user by asking about the user’s stage of CKD, stage of fibrosis, prior treatment, and comorbidities
3. Assess user knowledge and values for treatment choices
4. Help individuals use and consider information appropriate to their values and needs [see Fig. 1] by creating a summary page that displays values that users should discuss with their clinician before starting treatment

This web-based tool will not replace a conversation with a clinician; it will be used to prepare patients for visits with a clinician. This manuscript describes the process for developing the Project HELP decision aid and our study protocol for pilot testing the tool.

## Methods

The content for this decision aid will be based on literature reviews and guidance from a diverse expert advisory group consisting of three nephrologists, three hepatologists, and two patients. Clinicians represent six different practices in four US regions (West, Midwest, Northeast, and Southeast). The content will be written at a sixth-grade reading level. The preliminary content of the decision aid will be based on Kidney Disease: Improving Global Outcomes (KDIGO) guidelines [17], the joint American Association for the Study of Liver Disease- Infectious Disease Society of America’s [22] combined guidelines and graded evidence, and our advisory board feedback.

First, the tool will assess individual factors that could impact HCV and CKD outcomes such as CKD severity, HCV genotype, stage of fibrosis, prior treatment history, and comorbidities. Based upon the answers to each question, the tool will provide tailored information and feedback to patients regarding these outcomes. Next, patients will be given a brief overview of HCV, CKD, the health effects of both diseases, and treatment options for both HCV and CKD. The decision aid will then review users’ knowledge of the content they received by asking eight review questions, providing immediate feedback as they answer the questions. Patients will rate what matters most to them as they weigh their treatment decisions. Patients will be able to select or enter questions to ask a provider about HCV and CKD. A summary page will be generated at the end of the participant’s session that can be saved or printed to access when talking to a clinician. The tool will follow International Patient Decision Aids Standards guidelines for decision tool development across applicable sections [33]. The tool will be developed over the course of 6–10 months and will include evidence synthesis, expert advisory board review, patient advisory review, readability assessment to ensure comprehension across literacy levels, and pilot testing sections of the tool for usability prior to the study beginning.

**Fig. 2:**
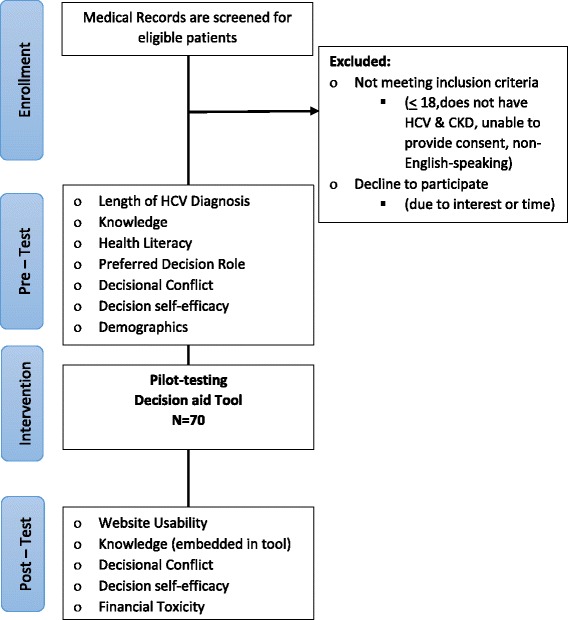
Aim 2—pilot-test study flow diagram

A pilot study of the tool will include 70 individuals. Eligibility criteria will include those who read and understand English, who are at least 18 years old and have a diagnosis of HCV (any genotype) and CKD of any stage. Recruitment will occur from October 2017 to May 2018.The research coordinator will screen medical records for eligible patients in our CKD clinics, dialysis units, and transplant clinics. Participants will have the choice to complete the study surveys using a web or paper-based version depending on their preference. The participant will be able to complete the study in person on a computer or tablet provided by us or at home. Participation requires approximately 30 min. Participants will receive a $20 gift card as compensation for their time. Figure 2 shows a study flow chart; we will follow MSD’s Guidelines for Publication of Clinical Trials in the Scientific Literature. This study is approved by the Human Research Protection Office (HRPO) at Washington University in St. Louis.

### Measures

Our primary outcome measures will include patients’ HCV and CKD knowledge, decisional conflict, decision self-efficacy, and the decision aid’s usability. To measure patients’ knowledge, we will develop items based on information that is considered vital to making treatment decisions, understanding what is HCV and CKD, the health effects of both diseases, and understanding facts that differentiate treatment options [34]. The response options will include true/false/unsure. The total number of correct responses will be calculated, with 1 point given for each correct response and 0 for unsure or incorrect responses. We will pilot test the items with patients before and after the study. We will calculate Cronbach’s alpha to assess the internal consistency of our questions.

To assess decisional conflict, we will administer the 4-item SURE Test version of the Decisional Conflict Scale (DCS) [35]. This scale measures whether individuals feel they have enough information to make a choice, are clear about their values for risks and benefits of their choice, and feel they have enough support to make a choice. Items will be scored as 1 (yes) or 0 (no).

To measure decision self-efficacy, we will use the lower literacy version of the Decision Self-Efficacy Scale [36]. This scale is a validated measure of an individual’s self-confidence or belief in their ability to make a decision. Individuals will be asked to rate on a three-item scale how confident they feel taking actions involved in making an informed choice (e.g., gathering information, asking questions, and expressing opinions). This scale has high levels of internal consistency (mean Cronbach’s alpha = 0.86) [28] and correlates with feeling informed, supported, and knowledgeable about decisions.

To assess usability, we will use the 10-item System Usability Scale (SUS) [37], a reliable, validated scale for evaluating the usability of our website. Secondary outcomes and potential covariates include factors that could influence decisions and quality of life such as clinical characters (e.g., CKD severity, stage of liver fibrosis, HCV genotype, prior treatment history of HCV and length of diagnosis, and comorbidities), demographics, health literacy [38], patients’ preferred decision role, and financial toxicity [39].

### Data analysis plan

Descriptive statistics will be calculated for all variables. Data will be examined for within group differences in outcomes (knowledge, decisional conflict, decision self-efficacy) among patients pre- and post-use of the DA in a multivariable linear regression model controlling for up to 5 independent covariates (e.g., age, stage of liver fibrosis, health literacy, CKD stage, and prior history of HCV treatment). We will include knowledge, decisional conflict, and decision self-efficacy as primary outcomes in three separate multivariable models, controlling for age and stage of liver fibrosis. Health literacy will be included as a covariate in the model with knowledge as an outcome. We will explore whether differences exist based upon CKD stage by including it as a covariate in our analyses. Prior history of HCV treatment will be included as a covariate in the model with knowledge as an outcome.

## Discussion

Many patients depend on their doctors to supply enough information to help facilitate treatment decisions. However, HCV and CKD treatment decisions for patients with both conditions are complex and can be overwhelming. Many patients want to participate actively in the shared decision-making process and be well-informed consumers and contributors of their own healthcare [40]. Decision aids can assist patients with understanding their choices, tailored to their specific needs and preferences. These tools can be used before, during, or after meeting with a clinician depending on clinic flow and patient preferences.

To our knowledge, tools that have been developed about HCV and CKD treatment choices have been for clinician use only. Our tool will be the first tool for patients and clinicians to use together that accounts for the unique needs of this population. After patients use our tool, they will be able to save or print a summary page that they can share and discuss with their clinicians during their visit to take a more active role in treatment decisions. Using this tool will potentially allow patients with chronic HCV infection and CKD to make an informed treatment decision and increase the rates of personalized treatment approaches.

**Fig. 3:**
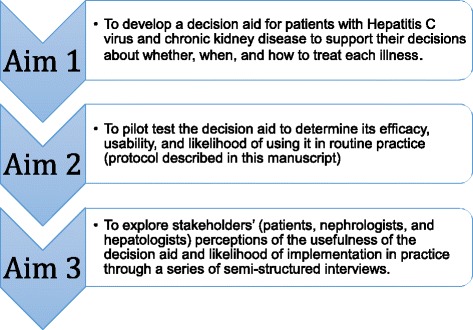
Project HELP—overall study objectives

Strengths of the study include personalized decision support tailored to patients’ clinical needs, the use of International Patient Decision Aids Standards guidelines for decision tool development, and the inclusion of an advisory group which consists of both clinicians and patients to help guide the development of the study and decision tool itself. Given that this is a pilot study, we will not know for sure whether our sample size is large enough to detect differences, but we selected our study design (pre-post within-subjects study design) and targeted sample size based on past studies demonstrating a small to medium effect size for most outcomes. After the study, we will conduct post-hoc power calculations to determine actual effect sizes to better plan for future, larger studies. This project is a single-site study with a moderate sample size, so results might not be generalizable. To address this, we plan to include a national sample of clinicians for our semi-structured qualitative interviews, which will be conducted as part of the next aim of this study (see Fig. 3).

Because there is no use of randomization, results should be interpreted as demonstrating the preliminary efficacy of the tool. We considered randomizing patients to the decision tool or a control group of usual care, but randomizing at the patient level could bias clinicians’ discussions with patients in the control or usual care group. Our pilot study is not equipped to conduct a multi-site trial randomizing at the provider or clinic level. This study can identify particular characteristics of individuals or groups that might experience challenges initiating treatment for HCV in the CKD population. This tool could provide a resource to facilitate patient-clinician discussions regarding HCV and CKD treatment options. We plan to conduct semi-structured qualitative interviews with both clinicians and participants after this pilot evaluation of the decision aid to gather feedback about implementing the tool into clinical practice beyond the duration of this proposal.

## Future plans

We will continue to work closely with our advisory board, patients, and clinicians to plan for subsequent studies and implementation. We will develop electronic communication strategies and disseminate our tool nationwide through multiple channels to maximize the usefulness of these findings in ongoing clinical practice and research efforts. Future larger studies could conduct a randomized trial comparing the tool to usual care, randomizing at the clinic level to account for the individualized approach clinicians may use when treating their patients.

## Abbreviations

CKD: Chronic Kidney Disease
DAAs: Direct-acting antivirals
FDA: Food and Drug Administration
HCV: Hepatitis C virus
SVR: Sustained virologic response
